# The Importance of Contextual Factors in Carrying Out Childhood Violence Surveys: a Case Study from Indonesia

**DOI:** 10.1007/s12187-017-9457-8

**Published:** 2017-02-17

**Authors:** Lauren Rumble, Ali Aulia Ramly, Mu’man Nuryana, Michael P. Dunne

**Affiliations:** 1 UNICEF Indonesia, World Trade Center 6, 10th Floor, Jl. Jenderal Surdirman Kav.31, Jakarta, 12920 Indonesia; 2 Ministry of Social Affairs of the Republic of Indonesia, Jalan Salemba Ryan, No.28, Jakarta, Pusat 10430 Indonesia; 3 School of Public Health and Social Work, Queensland University of Technology, Victoria Park Rd, Kelvin Grove, QLD 4059 Australia

**Keywords:** Indonesia, Prevalence survey, Violence against children, Survey response

## Abstract

Nationally representative research into violence against children is necessary to understand the scale and complexity of such violence and to evaluate prevention efforts. To date, however, most countries do not have adequate data. In 2013, the government of Indonesia conducted a national Violence Against Children Survey (VACS). This was a cross-sectional household survey of male and female 13-to-24- year-olds designed to estimate physical, emotional, and sexual violence prevalence. The target was to interview at least 2580 individuals; but response rates were much lower than anticipated (females = 66.6%; males = 56.1%). Insufficient data was available across several variables and there were unexpected anomalies in obtained data. We conducted a retrospective analysis of the survey to understand impediments and to advise future national efforts in Indonesia and other low-to-middle-income contexts. Survey managers and implementers (*n* = 22) were interviewed online and in person. We also carried out secondary analysis of the child survey data to identify factors possibly associated with (non-)response and assessed field notes from interviewers. Culturally inappropriate timing of data-collection (during Ramadan) may have had a negative impact on household responsiveness and the availability of children at home. Face-toface interviews in households were considered to impede participation and disclosure. Survey field staff and managers expressed the need for deeper training and a more comprehensive pilot. Recommendations to improve privacy and anonymity include the use of self-administered questionnaires and school-based rather than at-home surveys. These and other findings from this case study may be useful in planning future surveys in Indonesia and similar social and cultural contexts.

## Global Context

Violence in childhood has become a high-priority concern in recent international public policy. The United Nations’ new Sustainable Development Goals (SDGs) include ambitious targets obliging countries to eliminate all forms of violence against women and children (United Nations General Assembly 2015). The targets for protection of children reflect increased recognition by policymakers of the devastating personal, social, and economic costs of childhood violence, as evidenced in many studies worldwide (Ammerman et al. 1986; Browne and Finkelhor 1986; Jewkes et al. 2010; Moore et al. 2015; Norman et al. 2012; Polusny and Follette 1995).

Governments require reliable scientific data upon which to measure progress. However, this is still an emerging field. The first global study on violence against children (Pinheiro 2006) concluded that data on the nature and extent of and the responses to childhood violence were unavailable in many countries. Since then, significantly more research on violence against children has become available, including population-based surveys on multiple forms of childhood violence (Hovdestad et al. 2015). The quality of data has also increased in many countries (Hillis et al. 2016). Nevertheless, prevalence and other data remain lacking in most middle- and low-income countries (Veenema et al. 2015).

Collecting data on violence against children is difficult for various reasons, and there is no international consensus on preferred measurement instruments. Studies differ in their definitions, research tools, and methodologies (United Nations Children's Fund 2014a; 2016), making international comparisons difficult. The occurrence of violence tends to be under-estimated, as certain forms of violence are under-reported in most settings (World Health Organization 2013; 2014a). Further, national surveys seldom record child neglect and emotional violence (Finkelhor and Lannen 2014; Stoltenborgh et al. 2013). In addition, particularly in the case of sexual violence, persistent cultural and systemic barriers make disclosure difficult (Paine and Hansen 2002; Watts and Zimmerman 2002); children may be afraid to disclose, not know where to seek help, or blame themselves for the abuse (McElvaney et al. 2014). Gender may also influence disclosure, in different ways depending on social norms and cultural practices.

Recent systematic reviews from the Asia Pacific region confirm high variation in reported rates of child violence, as a result of differing measurement procedures and definitions (Fang et al. 2015a; Fry et al. 2012; United Nations Children's Fund 2012; 2014c). Major gaps remain in prevalence data from several countries; in particular, most low-income countries in the region have no data for neglect, emotional violence, or witnessing parental violence (Fang et al. 2015a). In all of Southeast Asia, there has been no published community-based survey that captures changes over time in the risk of child abuse. Thus, additional scientifically rigorous evidence is needed for this region to help governments take action (Dunne et al. 2015).

Like many complex social phenomena, violence against children and young people is difficult to study objectively. While social science research designs, techniques for representative sampling, self-report measurements, and survey procedures can be rigorously applied to yield valid findings about violence in communities, this standard is not always achieved. Research efforts that have been successful in some populations may encounter difficulties in other societies. When large-scale research projects do not achieve their purpose—when considerable amounts of human effort, goodwill, technical expertise, and funding are present but the outcomes are not sufficient—it is necessary to analyse what went wrong, and how future work can be improved.

In this case study, we examine a recent situation in Indonesia where a national-level VACS encountered several impediments and the outcomes were considered not to represent the true extent of the problem of violence against children in the country.

## The Setting

Indonesia is home to the largest Muslim population in the world and is an important political and economic power—the biggest economy in Southeast Asia and a member of the G20. Indonesia was prominent in intergovernmental discussions preceding the adoption of the SDGs in 2015, and has incorporated many of the targets into its own ‘Medium Term Development Plan 2015–2019’, including Target 16.2: to end all forms of violence against children (United Nations Economic and Social Council 2015; United Nations General Assembly 2015). Nevertheless, Indonesia still lacks rigorous data on all forms of violence against children, notwithstanding costly efforts and acknowledgement by the government of the importance of such data in national plans.

The limited data available point to a significant problem with violence against and amongst children in Indonesia. A 2007 survey in schools found physical violence amongst boys 13–15 years old to be nearly the highest in the world (Indonesia Ministry of Health et al. 2007). Domestic violence prevalence has been estimated to be as high as 60% in some rural areas and 25% in urban areas (Fulu et al. 2013). It is likely that a large number of children witness domestic violence at home or in the community. For some, it appears that such violence is normal; one study found that both girls (45%) and boys (48%) aged 15–19 believe domestic violence is sometimes justifiable (Statistics Indonesia (BPS) et al. 2013).

In response to these concerns, the Indonesian government mounted a national survey in 2013 to determine the magnitude of violence against children and to identify patterns of risk and protective factors, in order to develop more targeted, better-informed prevention programmes and policy initiatives (Alit et al. 2014). The study was a cross-sectional household survey of 13-to-24-year-old females and males on violence against children that used the VACS methodology (Reza et al. 2009). This is a major international effort which had been applied quite successfully in eight other low- and middle-income countries at the time of writing (Centers for Disease Control and Interuniversity Institute for Research and Development Comite de Coordination 2013; Reza et al. 2009; Steering Committee on Violence Against Children 2014; The Ministry of Gender Children Disability and Social Welfare of the Republic of Malawi 2014; United Nations Children’s Fund Nigeria and the US Centers for Disease Control and Prevention 2015; United Nations Children’s Fund et al. 2011; United Nation’s Childrens Fund et al. 2012; Vagi et al. 2016; Zimbabwe National Statistics Agency (ZIMSTAT) et al. 2013).

Across studies, the VACS methodology uses a common set of age ranges and definitions for sexual, physical, and emotional violence. Questionnaires are drawn from a range of international survey instruments (Sumner et al. 2015). In Indonesia, the government funded and led the VACS 2013; United Nations Children’s Fund (UNICEF) Indonesia and the US Centers for Disease Control and Prevention (US CDC) provided technical and operational support.

### Definitions

The study in Indonesia attempted to estimate lifetime and 12-month prevalence of physical, emotional, and sexual violence, in accordance with WHO definitions (Krug et al. 2002; World Health Organization 1999). *Sexual violence* was defined as sexual touching, attempted penetrative sex, physically forced sex, and pressured sex. *Physical violence* was defined as including punching, kicking, whipping, beating with an object, choking, smothering, trying to drown, burning intentionally, and/or using or threatening to use a weapon to hurt someone. *Emotional violence* was defined as verbal violence by parents or guardians (only), such as being told that one was unloved or that parents or guardians wished one were dead or had never been born, or being ridiculed or put down. Bullying, neglect, witnessing parental violence, exploitation, and other forms of child violence sometimes used in international definitions or studies from the region were not included (Fang et al. 2015a; World Health Organization 1999). Any respondent who answered ‘yes’ to any question on experiencing any form of violence was asked follow-up questions about his or her age at the time of the incident and details on the perpetrator, location, and time of day. *Children* were defined as individuals below 18 years of age, as stipulated in the *Convention on the Rights of the Child* (UN General Assembly, 20 November 1989), ratified by Indonesia in 1990. *Adolescents* were defined as individuals between 18 and 24 years, as defined by the World Health Organization (World Health Organization 2014b). The VACS received ethics approval from the Ministry of Health of Indonesia and the US CDC institutional review board in 2013.

### Methodology

To reflect Indonesia’s cultural, economic, and social diversity, the national statistics agency opted to collect data from five regions across the country. The study used a four-stage cluster sample survey design employing the Kish method to randomly select one individual per household for interview (Kish 1949). The study was carried out between July and August 2013. A target was set to achieve a sample size of 2580 completed interviews (645 females and 645 males for each of rural and urban settings). Inclusion criteria were age (13–24 years), fluency in Bahasa Indonesia, and residence in the household for at least six months. Interviews were conducted in the household, but interviewers were instructed to secure a private space. Data were collected amongst two age cohorts: ‘children’ aged 13 to 17 years and ‘adolescents’ aged between 18 and 24 years. Data were collected using electronic notepad devices, on which explanatory preambles and questions appeared and the interviewers recorded responses from the children and adolescents. Survey management was complex: social workers from the Indonesian Ministry of Social Affairs and researchers from the national statistics agency carried out the data collection. Overall survey management was led by a team comprised of the Ministry of Planning, the Ministry of Women’s Empowerment and Child Protection, and the Ministry of Social Affairs. UNICEF and the US CDC provided technical support and some funds for training.

### Indonesia VACS Response Rates and Results

#### Participation Rate

Based on experience with VACS surveys in other countries, including in the region (Steering Committee on Violence against Children 2014; Council for the Welfare of Children 2016), it was anticipated that the household and individual participation rates would be approximately 90%. As seen in Table 1, however, the achieved response rates (including both initial refusals and initial assent later withdrawn) were considerably lower. Households in urban areas were particularly unlikely to participate. When permission was given for the child to be approached, rural and urban males (at 54% and 58% respectively) were less likely to agree to participate in the survey than females (65% rural and 69% urban).

**Table 1:** Household and individual response rates by gender and location (urban v. rural). Based on data reported in the Indonesia VACS Report 2014 (Alit et al. (2014). *Ringkasan hasil: Survey kekerasan terhadap anak Indonesia 2013.* Jakarta: Government of Indonesia)

Household response rate	Females		Males	
	*n*	%	*n*	%
Urban	2482	73.8	3004	79.1
Rural	2372	84.5	2898	86.0
Total	4854	79.1	5902	82.5
Individual response rate	*n*	%	*n*	%
Rural	590	68.7	733	54.1
Urban	559	64.5	660	58.4
Total	1149	66.6	1393	56.1

Given that the achieved sample was less than optimal, the study has limited statistical power. Many cell sizes were too small to yield reliable prevalence estimates, and cross-tabulation to examine associations between variables (e.g., males and females who had or had not witnessed intimate partner violence at home) often was not viable.

#### Prevalence Estimates

Table 3 shows estimates of lifetime and past year prevalence for key indicators of violence presented in the government’s VACS Report. It must be emphasised here that these data cannot be cited as being valid indicators of the true occurrence of violence. Significant doubts about internal validity arise when comparing the apparent prevalence for younger and older cohorts. For sexual, physical, and emotional violence, self-reports were much higher amongst children aged 13 to 17 years than the older respondents. Some of the estimates lack face validity; for example, it is implausible that the true underlying risk of physical violence against older girls is less than one-third the level experienced by slightly younger girls; similarly, it is highly unlikely that the actual risk of emotional violence against older girls is only one-fifth the risk among younger girls.

## Lessons Learned from The Indonesia Study

### Methods and Materials

For this case study, we undertook a lessons-learned analysis of the implementation of the VACS study, including administration of an online survey of purposively selected key informants who had been directly involved in the VACS Indonesia. We utilised an online survey as our primary interview method to protect respondents’ privacy and promote participation. A convenience sample of 22 respondents could be located and surveyed in 2016, slightly less than three years after the main community survey was conducted. They had been in advisory, management or data collection roles for the survey: 14 were from government, three from UNICEF, two from the CDC, and three were child protection research experts from academia (Table 2). Most respondents (19) had had some professional research training or prior experience carrying out social surveys involving household interviews.

**Table 2:** Online survey participants by employment and role in the VACS Indonesia 2013

	Survey team member or team leader	Technical advisor	Research expert (not participating in the VACS design or administration)	Management (in government)
Government	10	2		2
Academic			3	
UN/CDC		5		

The online survey consisted of twenty open- and closed-ended questions. We conducted the survey in Indonesian and English languages. All data collected from the survey were non-identifiable.

In addition, we conducted in-person interviews with five people who were involved in and had detailed knowledge of the VACS Indonesia, from the CDC Indonesia, UNICEF Indonesia, and the Ministry of Social Affairs. We used a topic list that was adapted to the participant but drew from the same themes as the semi-structured questionnaire for the online survey. All interviews were conducted in English unless requested otherwise. Interview notes and transcripts were coded manually.

We also gained access to field notes from eight interviewers and survey managers that were taken contemporaneously as the VACS progressed. The notes included free form comments about issues that arose in trying to locate selected households, barriers encountered when approaching households, difficulties experienced when seeking permission from parents/guardians or in gaining consent from children and adolescents, logistical and operational challenges (for example difficulties using electronic notepads), and language barriers, among others.

We triangulated information from the online survey with the in-person interviews and field notes. Data were analysed iteratively. The authors jointly discussed the results of the interviews, online survey, and analysis of field notes and compared the results of the VACS Indonesia to those of other VACS from the region (Lao PDR, Cambodia, and the Philippines) for rigorous and systematic analysis. We developed a process narrative using the constant comparative method of analysis for policy-relevant qualitative research (Pope et al. 2000).

This case study obtained ethical approval from the Human Research Ethics Committee of the Queensland University of Technology (QUT). Free and informed consent was obtained from all participants, and care has been taken that no comments can be traced back to an individual.

## Findings

Half of the respondents said that household refusal to participate mainly occurred because heads of household were too busy or not interested. Some thought that the survey topic was felt by householders to be too sensitive to discuss, although it should be noted that the VACS was introduced to households as a general survey of children’s health and well-being. One of the respondents noted that: ‘Talking about violence against children is not an acceptable social norm in Indonesia’ and another that ‘in Indonesia, [there is] a common perception that violence is a private matter’.

There was little consensus among survey administrators as to why the response rate was lower among urban than rural households, although several commented that this might be attributable to ‘urban survey fatigue’ or to urban households being busier than rural ones. A couple of respondents at the management level suggested that people in rural households might feel less able to refuse to participate in a government-led survey. These respondents suggested that it is possible the residents were intimidated by the presence of government researchers. Analysis of the response rate tables in the datasets indicated that one of the reasons for lower than anticipated response rates was that a portion of households did not manage to complete the questionnaire (approximately 9% of female households and 7% of male households). Considering suggestions from key stakeholder interviewers and the daily field notes together, it appears that adult males (fathers, grandfathers or other significant male adults) were most likely to refuse to allow the child or adolescent to participate.

About a third of survey staff suggested the main reason for individual refusal by children and adolescents was the sensitive subject matter. They observed that it was difficult for respondents to speak openly on personal matters with strangers. Survey staff posited that this might be due to lack of privacy for interviewees, and fear children may have felt discussing acts their parents or relatives may have committed in the home setting where the interviews took place. Some felt the interview field staff were insufficiently trained to create rapport with children and adolescents. Other reasons suggested for low individual response rate (or incomplete interviews that were commenced) included inadequate explanation to children of the survey’s purpose, and some respondents becoming bored by the length of the interview. It was also suggested that children and young people in Indonesia are not accustomed to being asked their opinions on sensitive personal and social problems. Field notes from some interviewers recorded their impressions that children had difficulty disclosing their experiences and talking about ‘private matters’.

Male children and adolescents were less likely than females to agree to be interviewed in the survey. Respondents were not sure why this was the case. About one-third of the key stakeholder respondents indicated that usually females would be expected to be less confident in their ability to say no to participating in such a survey. About a quarter of the survey staff respondents noted that Indonesian males might be less willing to participate in discussions on violence and particularly on sexual violence for socio-cultural reasons. This may have been more likely if there was gender mismatch between young male respondents and the interviewer.

Most survey staff mentioned that the timing of the survey during Ramadan (a major religious holiday period) affected individual and household participation. Ramadan coincides with school holidays in Indonesia, when many children stay with their grandparents or extended family. Over half of the survey staff said they had not anticipated this problem. Survey managers described being more concerned at the time to avoid the rainy season, which would have limited access to a number of rural sites. Further, analysis of field notes indicated that interviewers had to mark many questionnaires ‘incomplete’ because, even though the child may have been willing to participate, they were alone at home at the time, and therefore the requirement of a guardian’s presence was not met. About half of the survey staff said they had some difficulty confirming that eligible respondents had been living in the household for six months.

Most respondents indicated that conducting the interviews only in Bahasa Indonesia restricted overall participation. Whilst Bahasa Indonesia is the one official national language in Indonesia, more than 700 other languages are spoken in the country, and the sampling sites included many regions and ethnic groups.

When interviewing the former survey staff, we discussed with them the basic findings regarding prevalence (Table 3). The respondents were asked to suggest reasons why there was such a large discrepancy between prevalence estimates for children (aged between 13 and 17 years) and adolescents (aged between 18 and 24 years). Only some staff suggested possible reasons, which included the fact that older participants were more likely to be married and less likely to consider sexual or other forms of violence as violence if committed by a partner; possible bias by interviewers due to differences in the ways they asked questions of children compared to older respondents; and recall bias by older respondents, where they could not accurately recall what happened during their childhood.

**Table 3:** Percentage of respondents who reported experiencing physical, emotional, and sexual violence and witnessing domestic violence in the Cambodia and Indonesia VACS. Based on data reported in the Indonesia VACS Report 2014 (Alit et al. (2014). *Ringkasan hasil: Survey kekerasan terhadap anak Indonesia 2013*. Jakarta: Government of Indonesia) and the Cambodia VACS Report 2013 (Steering Committee on Violence Against Children. (2014)). *Findings from Cambodia’s Violence Against Children Survey (VACS) 2013*. Phnom Penh: UNICEF

Indonesia VACS					Cambodia VACS				
Percentage of respondents who reported experiencing any form of physical violence					Percentage of respondents who reported experiencing any form of physical violence				
Age	Females		Males		Age	Females		Males	
	*n*	% (95% CI)	*n*	% (95% CI)		*n*	% (95% CI)	*n*	% (95% CI)
18–24 years	331	8.0 (4.4–11.5)	313	40.7 (34.6–46.9)	18–24 years	599	52.7 (47.2–58.1)	613	54.2 (49.4–59.0)
13–17 years	429	26.7 (20.8–32.6)	450	51.7 (45.8–57.6)	13–17 years	522	61.1 (55.9–66.2)	642	58.2 (53.1–63.3)
13–17 years (in the past 12 months)	429	11.8 (8.0–15.5)	449	29.1 (23.7–34.4)	13–17 years (in the past 12 months)	522	15.3 (11.7–18.9)	642	12.5 (9.2–15.8)
Percentage of respondents who reported experiencing any form of emotional violence by a caregiver					Percentage of respondents who reported experiencing any form of emotional violence by a caregiver				
Age	Females		Males		Age	Females		Males	
	*n*	% (95% CI)	*n*	% (95% CI)		*n*	% (95% CI)	*n*	% (95% CI)
18–24 years	329	3.8 (0.9–6.7)	307	13.4 (7.8–18.9)	18–24 years	598	19.4 (15.8–23.0)	609	25.0 (20.6–29.4)
13–17 years	429	18.5 (13.8–23.3)	451	21.3 (16.6–26.0)	13–17 years	522	24.3 (19.2–29.4)	640	27.3 (23.0–31.6)
13–17 years (in the past 12 months)	429	9.4 (6.2–12.6)	450	12.6 (9.2–15.9)	13–17 years (in the past 12 months)	522	9.7 (6.5–12.8)	642	9.6 (6.8–12.4)
Percentage of respondents who reported witnessing a parent being punched, kicked, or beaten					Percentage of respondents who reported witnessing a parent being punched, kicked, or beaten				
Age	Females		Males		Age	Females		Males	
	*n*	% (95% CI)	*n*	% (95% CI)		*n*	% (95% CI)	*n*	% (95% CI)
18–24 years	330	10.6 (6.5–14.7)	310	12.3 (8.2–16.4)	18–24 years	598	15.3 (11.8–18.8)	608	18.1 (14.2–21.9)
13–17 years	429	24.5 (18.8–30.2)	448	24.4 (19.6–29.1)	13–17 years	522	20.6 (16.5–24.7)	642	24.5 (20.1–28.9)
13–17 years (in the past 12 months)	426	9.0 (5.4–12.7)	434	9.7 (6.0–13.4)	13–17 years (in the past 12 months)	116	37.4 (27.4–47.3)	150	34.7 (26.7–42.7)
Percentage of respondents who reported experiencing any form of sexual violence					Percentage of respondents who reported experiencing any form of sexual violence				
Age	Females		Males		Age	Females		Males	
	*n*	% (95% CI)	*n*	% (95% CI)		*n*	% (95% CI)	*n*	% (95% CI)
18–24 years	331	6.3 (3.1–9.5)	311	6.4 (3.4–9.4)	18–24 years	599	4.4 (2.5–6.3)	613	5.6 (3.5–7.7)
13–17 years	427	9.0 (5.6–12.3)	437	11.0 (7.6–14.4)	13–17 years	522	6.4 (3.7–9.0)	642	5.2 (3.1–7.3)
13–17 years (in the past 12 months)	427	4.1 (2.4–5.8)	437	8.3 (5.4–11.2)	13–17 years (in the past 12 months)	518	3.0 (1.0–5.0)	639	0.1 (0.0–0.2)

Due to time pressures, a full pilot in both rural and urban locations was not conducted. Limited time and budget were made available for the training of researchers, and many researchers were required to use electronic notepads in data collection for the first time. There was significant concern expressed by the respondents about the quality of staff training and data collection. The majority of survey staff said that the training for team leaders and interviewers was inadequate. It was noted that training for survey managers was done in English with some simultaneous translation into Bahasa Indonesia. Respondents recommended that in future, local child protection research experts should conduct comprehensive training entirely in Bahasa Indonesia. About a quarter of survey staff respondents said that interviewers and field data collection managers should have more prior experience conducting social surveys related to child protection or other sensitive public health concerns (for example, HIV and sexual behaviour). Gender matching of enumerators to child respondents was also recommended by most survey staff. Although this condition was originally recommended for the main survey, field notes and interviewed staff indicated that it was not rigorously applied.

Several significant technical issues emerged from the use of electronic notepads for data collection. Half of the survey staff said it affected the results, for example not being able to conduct interviews as planned. There was perceived to be inadequate training in the use of the notepads, and some interviewers lacked confidence applying this technology in their fieldwork. The notepads were important for this survey to standardise the interviews, but unfortunately, late arrival of the equipment in Indonesia delayed the pilot survey, which then pushed back the main survey into the Ramadan period. In the in-depth interviews, some respondents suggested that interviewers should have been fully prepared to use electronic notepads for data collection (for example, planning for lack of internet access at some sites, battery failure and occasional unreliable electricity access, or software problems). Nearly half of the staff survey respondents recommended that the management of future surveys be made more sensitive to the environmental conditions in which such surveys are done.

A few administrators suggested it was better to conduct the survey at schools instead of homes, while some also suggested the interview mode could include both face-to-face interviews and self-administered questionnaires. In Indonesia, especially in rural areas, most households are not private spaces; they have few or no separate rooms. Self-administered questionnaires may thus be more appropriate to the Indonesian context, so that it is not necessary to verbalise responses to questions about violence.

To further understand the findings from the VACS Indonesia, including the lower than anticipated response rates, we compared results with the VACS conducted in Cambodia, which was published officially in 2014 (Table 3). Lifetime and past year rates of sexual violence, for example, are higher amongst boys compared to girls in certain age groups: for boys aged 13–17 years in Indonesia and for boys aged 18–24 years in Cambodia. This apparent similar or higher risk for boys compared to girls has also been observed in studies from other countries in the region (Choo et al. 2011; Fang et al. 2015b; Finkelhor et al. 2013). Special efforts should be made to further investigate why boys are reporting higher rates than girls. These findings from Indonesia and Cambodia are the converse of results from VACS conducted in Africa and Haiti, and it is not clear whether they are indicative of higher prevalence or whether it is that boys are more confident to report.

Comparison of some of the other main violence indicators measured by the VACS is also presented in Table 3, within 95% confidence intervals. Notable are the much higher reported rates of physical violence in Cambodia as compared to Indonesia. More than half of girls (61.1%) aged 13–17 years reported having experienced any form of physical violence in Cambodia, as compared to less than a third of girls in Indonesia for this same age range, and only 8.0% (4.4–11.5) of girls aged 18–24 years. These estimates of physical violence are also much lower than found in other studies in Indonesia with significantly higher individual response rates (Bhatla et al. 2015; Indonesia Ministry of Health et al. 2007).

Lifetime and past year prevalence of all forms of violence reported here are higher for all age groups, for both boys and girls, in the Cambodia VACS. In addition, almost 40% of girls (37.4%, 24.4–47.3) and 34.7% (26.7–42.7) of boys had witnessed a parent being punched, kicked, or beaten in the 12 months preceding the survey. For Indonesia, only 9.0% of girls (5.4–12.7) and 9.7% of boys (6.0–13.4) in this age range said they had witnessed parental violence. Whilst there are no national prevalence data on domestic violence in Indonesia, the findings from various localised studies in both western and eastern parts of the country indicate relatively high levels of this kind of violence (Fulu et al. 2013), which are at odds with the low prevalence of witnessing domestic violence derived from the VACS survey of young people.

For both Indonesia and Cambodia, prevalence of emotional and physical violence, as well as witnessing any form of violence in the community, were highest for boys and girls between 13 and 17 years. It is possible that this age group is more likely than others to disclose instances of violence in surveys of this nature, but more analysis is needed. It is notable that the Indonesia data show significantly lower estimates of all forms of violence amongst the older age groups compared to the 13–17 years age range, for both boys and girls. The differences for girls are very substantial, yet the two cohorts differ in age by only a few years. The age group differences in the Cambodia VACS are much smaller. This may point to problems during data collection, such as inconsistency among interviewers in the time reference periods given for questions (for example, whether they asked respondents to reflect on their childhood for younger respondents, but perhaps more recent time periods for older respondents). Unfortunately, the qualitative interviews did not identify clear reasons for this significant anomaly.

## Discussion

To the best of the authors’ knowledge, this analysis is the first effort to document and learn from the experience of conducting a nationally representative survey on childhood violence in Southeast Asia. Its main purpose is to provide insights and practical suggestions for future research efforts, especially in Indonesia. It is also the first internationally published academic article about the VACS in Indonesia. Until now, the VACS Report has only been available in Bahasa Indonesia and no data extracted from it have been published externally. Despite concerns about the representativeness and quality of the data, the government made the pragmatic decision to utilise the main prevalence estimates in forward planning for child protection, recognising that the data are indicative of the scale of the problem, although not conclusive. The data in Table 3 were included in the country’s National Medium Term Strategic Plan. This allowed the government to justify increased investment in this area (for instance, the Ministry of Women’s Empowerment and Child Protection’s budget quadrupled in 2016). The decision to use the data in this way also honours the courage and contribution of the children and adolescents who volunteered to participate. However, as stated above, all partners recognised that the study was not sufficiently rigorous, and there is a need for better scientific evidence on childhood violence in Indonesia.

This case study identifies at least five areas for reform in future research efforts in Indonesia and similar sociocultural contexts. These recommendations all emphasise the importance of careful planning prior to data collection.

First, researchers and supporting institutions should pay attention to timing. Major religious and other holidays should be avoided. Ramadan, in particular, is a fasting month for Muslims. As in 2013, Ramadan can coincide with school holidays, altering the availability and perhaps willingness of householders and children to volunteer for surveys of this nature.

Second, the confidentiality of young people’s interviews should be prioritised in social contexts where individual privacy is not common in the household. Self-administered questionnaires administered at school, for example, may be more appropriate to Indonesia. Experiences related to sexuality, and especially sexual transgressions, are sensitive matters for individuals, families, and communities. This may limit open discussion and disclosure of violence, especially sexual violence or parent-on-parent violence. Global studies on violence against women have found that anonymous self-administered questionnaires result in higher reporting rates than interviewer-administered questionnaires for these reasons (García-Moreno et al. 2005). Although intimate partner violence may be fairly common in Indonesia, it is often not disclosed for fear of disrupting household harmony (Hakimi et al. 2001). A study from Indonesia’s Aceh province described this reluctance to disclose or intervene in family violence as *nafsi nafsi*—roughly translated, ‘your business is yours and you have your own solution’ (Stark et al. 2012). Violence perpetrated against women and girls is seriously under-reported and may be normalised and rationalised in some communities, including women and girls themselves (Hayati et al. 2013; Hayati et al. 2011). Similarly, neighbours of domestic violence victims may ‘not want to know’ about sexual or other violence that women and adolescent girls experience (Horn, *Piloting the neighbourhood method to gather information on the prevalence of child protection concerns in Indonesia*, unpublished report, 2011).

Furthermore, a school-based self-administered survey may have the advantage of involving children living in Islamic boarding schools or other residential care institutions, a common practice in Indonesia (Center for Child Protection, University of Indonesia (PUSKAPA) UI 2014). Such surveys may also better protect children (Havermans et al. 2015) as well as increase participation. Unfortunately, some vulnerable children, such as those not in school, child migrants or living on the street, will continue to be invisible to household- and school-based surveys. Complementary, targeted studies are recommended to capture experiences of violence experienced among these marginalised groups (Rubenstein and Stark 2016).

Third, investments should be made to develop research capability in the field of child protection for survey teams. The SDGs focused on elimination of violence against children and women are laudable goals, but the measurement of progress will depend on having teams of researchers and program evaluators in key government departments and universities with advanced skills in the design, implementation, and analysis of violence studies. An important element, as shown in this lessons learned analysis, will be comprehensive training in local languages for interviewers and field survey managers in how to establish and maintain rapport with children who may be feeling fear and shame. Survey teams should include trained counsellors, for example. Devries and colleagues make several practical suggestions in relation to these matters, especially for interviewer selection and training (Devries et al. 2015, 2016) as do others (Currie and Heykoop 2012; Graham et al. 2013; Schenk and Williamson 2005; United Nations Children's Fund 2015).

The fourth recommendation is for tighter operational management protocols. The low individual response rates, data entry errors, and the implausible variance between age cohorts found here (especially for females) indicates a strong need to ensure that interviews are conducted in standardised ways. Communication between team leaders and enumerators is vital to identify non-response or refusal patterns early on. Ideally, these sources of error should be minimised by more comprehensive pilot development work.

Finally, it may be necessary to adapt the VACS questionnaire. The internationally applied interview does not include questions on neglect, a common form of child maltreatment in the Asia Pacific region especially amongst affluent urban households (Fang et al. 2015a). To address these concerns, first, a review of the questionnaire by children and adolescents, as well as local researchers, is needed. Several respondents who managed the 2013 survey suggested the introduction of a short questionnaire to assess prevalence amongst a larger sample, accompanied by a longer questionnaire for in-depth interviews on risk and protective factors as well as perpetrators with a smaller sample. As one of the online survey respondents noted in our case study: ‘More attention should be spent to understand how children and young people disclose incidents of violence. Insufficient attention was paid to the diversity of cultures in the fourth most populous country in the world. International tools and approaches need to be adapted’.

In the Philippines, such a mixed-methods approach was used recently and yielded higher response rates at household and individual levels (Council for the Welfare of Children 2016). In Lao PDR, VACS researchers included a short questionnaire for participants to complete anonymously at the end of the interview. Significantly higher reports of sexual violence amongst females were reported. For example, 10.2% of females disclosed sexual touching and 6.2% of females disclosed unwanted attempted sex using anonymous reporting, compared to 6% and 1.4% respectively. This means that females were 70% more likely to report unwanted sexual touching and 343% (more than four times) more likely to report unwanted attempted sex using the anonymous method (United Nations Children’s Fund, Violence against Children Survey in Lao PDR: Alternative methodology for capturing violence data, p. 2, unpublished report 2016).

There are several limitations to the present analysis. First, it may not fully represent the views of all involved because only a convenience sample of key stakeholders could be contacted and engaged nearly three years after the main survey. Second, the analysis of field notes was not exhaustive, as field notes were not available for many enumerators. Third, the secondary data analysis was limited to just age and gender because of concerns that further disaggregation of data (for example, by location, family type, parental characteristics) would have low statistical power and might misrepresent the true situation of demographic subgroups.

## Conclusion

Rigorous research is needed to measure progress toward the SDG targets on violence eradication. The Indonesian government has committed budget and resources to fund another prevalence survey in 2018 in its efforts to address violence against children. To do so will require careful reflection on previous research efforts and on the selection of better methods.

There is no gold standard measure of violence against children. For countries like Indonesia, a possible future approach is to adopt the best characteristics of the VACS interview along with elements from complementary tools, such as the ISPCAN Child Abuse Screening Tool (ICAST), the WHO Adverse Childhood Experiences (ACE) International Questionnaire, and the Juvenile Victimisation Questionnaire (JVQ) (Dunne et al. 2009; Finkelhor et al. 2015; Ramiro et al. 2010; Runyan et al. 2009; Zolotor et al. 2009). There is a need for a systematic comparison of these tools for particular social contexts and survey modes. There may also be substantial benefit from research that uses ‘big data’ surveys embedded in social media platforms, which may be appropriate to young Indonesians. Increased opportunities for practical information exchange between governments, development practitioners, and academic researchers are needed to share examples of new and emerging areas of good practice. Prior to embarking on future surveys, Indonesia should therefore consider designing and carefully pilot-testing a questionnaire survey mode better suited to the context, for example the use of self-administered electronic or paper-based questionnaires. Based on the very high response rates (>90%) for the recent Global School-based Student Health Survey (World Health Organisation, *Global School-based Student Health Survey*, unpublished report, 2015), involving 11,142 male and female students aged 13–17 years, a self-administered school-based survey may be preferable. To reduce non-completion of the questionnaire or refusal to participate, children from demographically diverse groups should be actively involved in reviewing and pilot-testing future questionnaires.

Organisations offering governments technical support for violence research have also noted that research methodologies remain fragmented and that no clear guidelines are in place in this regard (United Nations Children's Fund 2014b). In the Asia Pacific region, Lao PDR and Cambodia have both adopted the standard VACS methodology, whereas the Philippines has developed its own tools, derived from both ICAST and VACS. This is in keeping with some studies that have found measures of child violence developed in Europe or the US ill suited to Asian contexts (Teimouri et al. 2015). Globally, childhood violence has become more visible in the international human rights, justice, and development agendas, but research and evidence in these areas are still emerging. This lessons-learned exercise represents one of the first attempts to reflect upon, critically analyse, and publicly share the results of a national research process in this field to inform evolving research efforts in Indonesia and abroad.

## References

[CR1] Alit K, Wismaayanti Y, Irmayani H, Widodo N, Susantyo B, Irwanto, Praptorahoarjo G (2014). Ringkasan hasil: Survey kekerasan terhadap anak Indonesia 2013 [Summary results: Violence against Children Survey Indonesia 2013].

[CR2] Ammerman RT, Cassisi JE, Hersen M, Van Hasselt VB (1986). Consequences of physical abuse and neglect in children. Clinical Psychology Review.

[CR3] Bhatla N, Achyut P, Khan N, Walia S (2015). *Are schools safe and gender equal spaces? Findings from a baseline study of school related gender-based violence in five countries in Asia*.

[CR4] Browne A, Finkelhor D (1986). Impact of child sexual abuse: a review of the research. Psychological Bulletin.

[CR5] Center for Child Protection, University of Indonesia (2014). Understanding vulnerability: a study on situations that affect family separation and the lives of children in and out of family care.

[CR6] Centers for Disease Control (CDC), Interuniversity Institute for Research and Development, and Comité de Coordination. (2013). *Violence against children in Haiti: findings from a national survey 2012.* Port-au-Prince: CDC.

[CR7] Choo W, Dunne MP, Marret M, Fleming M, Wong Y (2011). Victimisation experiences of adolescents in Malaysia. Journal of Adolescent Health.

[CR8] Council for the Welfare of Children (CWC) (2016). National baseline study on violence against children in the Philippines.

[CR9] Currie V, Heykoop C (2012). Child and youth centred accountability: a guide for involving young people in monitoring and evaluation of child protection systems.

[CR10] Devries K, Child J, Elbourne D, Naker D, Heise L (2015). “I never expected that it would happen, coming to ask me such questions”: ethical aspects of asking children about violence in resource poor settings. Trials.

[CR11] Devries K, Naker D, Monteath-van Dok A, Milligan C, Shirley A (2016). Collecting data on violence against children and young people: need for a universal standard. International Health..

[CR12] Dunne M, Zolotor A, Runyan D, Andreva-Miller I, Choo W, Dunne S (2009). ISPCAN child abuse screening tools retrospective version (ICAST-R): Delphi study and field testing in seven countries. Child Abuse and Neglect.

[CR13] Dunne M, Choo W, Madrid B, Subrahmanian R, Rumble L, Blight S, Maternowska M (2015). Violence against children in the Asia Pacific region: the situation is becoming clearer. Asia-Pacific Journal of Public Health.

[CR14] Fang X, Fry D, Brown D, Mercy J, Dunne M, Butchart A (2015). The burden of child maltreatment in the East Asia and Pacific region. Child Abuse and Neglect.

[CR15] Fang X, Fry D, Ji K, Finkelhor D, Chen J, Lannen P, Dunne M (2015). The burden of child maltreatment in China: a systematic review. Bulletin of the World Health Organization.

[CR16] Finkelhor D, Lannen P (2014). Dilemmas for international mobilization around child abuse and neglect. Child Abuse and Neglect.

[CR17] Finkelhor D, Ji K, Mikton C, Dunne M (2013). Explaining the lower rates of sexual abuse in China. Child Abuse and Neglect.

[CR18] Finkelhor D, Turner HA, Shattuck A, Hamby SL (2015). Prevalence of childhood exposure to violence, crime, and abuse: results from the national survey of children’s exposure to violence. JAMA Pediatrics.

[CR19] Fry D, McCoy A, Swales D (2012). The consequences of maltreatment on children’s lives: a systematic review of data from the East Asia and Pacific region. Trauma, Violence & Abuse.

[CR20] Fulu E, Jewkes R, Roselli T, García-Moreno C (2013). Prevalence of and factors associated with male perpetration of intimate partner violence: findings from the UN multi-country cross-sectional study on men and violence in Asia and the Pacific. The Lancet Global Health.

[CR21] García-Moreno C, Jansen H, Watts C, Ellsberg M, Heise L (2005). WHO multi-country study on women’s health and domestic violence against women: initial results on prevalence, health outcomes and women’s responses.

[CR22] Graham A, Powell M, Taylor N, Anderson D, Fitzgerald R (2013). Ethical research involving children (ERIC).

[CR23] Hakimi M, Hayati E, Marlinawati V, Winkvist A, Ellsberg M (2001). Silence for the sake of harmony: domestic violence and women’s health in central java, Indonesia.

[CR24] Havermans N, Vanassche S, Matthijs K (2015). Methodological challenges of including children in family research: measurement equivalence, selection bias and social desirability. Child Indicators Research.

[CR25] Hayati E, Högberg U, Hakimi M, Ellsberg M, Emmelin M (2011). Behind the silence of harmony: risk factors for physical and sexual violence among women in rural Indonesia. BMC Women's Health.

[CR26] Hayati E, Eriksson M, Hakimi M, Högberg U, Emmelin M (2013). “Elastic band strategy”: Women’s lived experiences of coping with domestic violence in rural Indonesia. Global Health Action.

[CR27] Hillis S, Mercy J, Amobi A, Kress H (2016). Global prevalence of past-year violence against children: a systematic review and minimum estimates. Pediatrics.

[CR28] Hovdestad W, Campeau A, Potter D, Tonmyr L (2015). A systematic review of childhood maltreatment assessments in population-representative surveys since 1990. PloS One.

[CR29] Indonesia Ministry of Health, Indonesia Ministry of Education, World Health Organization, & Centers for Disease Control and Prevention (2007). Global school-based student health survey (GSHS) Indonesia.

[CR30] Jewkes R, Dunkle K, Nduna M, Jama P, Puren A (2010). Associations between childhood adversity and depression, substance abuse and HIV and HSV2 incident infections in rural south African youth. Child Abuse and Neglect.

[CR31] Kish J (1949). A procedure for objective respondent selection within the household. Journal of the American Statistical Association.

[CR32] Krug E, Mercy J, Dahlberg L, Zwi A (2002). The world report on violence and health. The Lancet.

[CR33] McElvaney R, Greene S, Hogan D (2014). To tell or not to tell? Factors influencing young people’s informal disclosures of child sexual abuse. Journal of Interpersonal Violence.

[CR34] Ministry of Gender, Children, Disability and Social Welfare of the Republic of Malawi (2014). Violence against children and young women in Malawi: findings from a national survey 2013.

[CR35] Moore, S., Scott, J., Ferrari, A., Mills, R., Dunne, M., Erskine, H., et al. (2015). Burden attributable to child maltreatment in Australia. *Child Abuse and Neglect*, 208–220. doi:10.1016/j.chiabu.2015.05.006. 10.1016/j.chiabu.2015.05.006 26056058

[CR36] Norman R, Byambaa M, De R, Butchart A, Scott J, Vos T (2012). The long-term health consequences of child physical abuse, emotional abuse, and neglect: a systematic review and meta-analysis. PLoS Medicine.

[CR37] Paine M, Hansen D (2002). Factors influencing children to self-disclose sexual abuse. Clinical Psychology Review.

[CR38] Pinheiro PS (2006). World report on violence against children: secretary-General’s study on violence against children.

[CR39] Polusny M, Follette V (1995). Long-term correlates of child sexual abuse: theory and review of the empirical literature. Applied and Preventive Psychology.

[CR40] Pope C, Ziebland S, Mays N (2000). Analysing qualitative data. BMJ.

[CR41] Ramiro L, Madrid B, Brown D (2010). Adverse childhood experiences (ACE) and health-risk behaviors among adults in a developing country setting. Child Abuse and Neglect.

[CR42] Reza A, Breiding M, Gulaid J, Mercy J, Blanton C, Mthethwa Z (2009). Sexual violence and its health consequences for female children in Swaziland: a cluster survey study. Lancet.

[CR43] Rubenstein B, Stark L (2016). A forgotten population: estimating the number of children outside of households in Cambodia. Global Social Welfare.

[CR44] Runyan D, Dunne M, Zolotor A (2009). Introduction to the development of the ISPCAN child abuse screening tools. Child Abuse and Neglect.

[CR45] Schenk K, Williamson J (2005). Ethical approaches to gathering information from children and adolescents in international settings: guidelines and resources.

[CR46] Stark L, Bancroft C, Cholid S, Sustikarini A, Meliala A (2012). A qualitative study of community-based child protection mechanisms in Aceh, Indonesia. Vulnerable Children and Youth Studies.

[CR47] Statistics Indonesia (BPS), National Population and Family Planning Board, Indonesian Ministry of Health, & ICF International (2013). Indonesia demographic health survey 2012.

[CR48] Steering Committee on Violence Against Children (2014). Findings from Cambodia’s violence against children survey (VACS) 2013.

[CR49] Stoltenborgh M, Bakermans-Kranenburg MJ, IJzendoorn MH (2013). The neglect of child neglect: a meta-analytic review of the prevalence of neglect. Social Psychiatry and Psychiatric Epidemiology.

[CR50] Sumner SA, Mercy JA, Saul J, Motsa-Nzuza N, Kwesigabo G, Buluma R, Marcelin LH (2015). Prevalence of sexual violence against children and use of social services - seven countries, 2007–2013. MMWR. Morbidity and Mortality Weekly Report.

[CR51] Teimouri M, Hassan MS, Griffiths M, Benrazavi SR, Bolong J, Daud A, Adzharuddin NA (2015). Assessing the validity of western measurement of online risks to children in an Asian context. Child Indicators Research.

[CR52] United Nations Children's Fund (2012). Child maltreatment: prevalence, incidence and consequences in the East Asia and Pacific region.

[CR53] United Nations Children's Fund (2014). Hidden in plain sight: a statistical analysis of violence against children.

[CR54] United Nations Children's Fund (2014). Measuring violence against children: inventory and assessment of quantitative studies.

[CR55] United Nations Children's Fund (2014). Violence against children in East Asia and the Pacific: a regional review and synthesis of findings.

[CR56] United Nations Children's Fund (2015). UNICEF procedure for ethical standards in research, evaluation, data collection and analysis.

[CR57] United Nations Children's Fund. (2016). Violence against children national surveys-East Asia and Pacific Regional Roundtable. Retrieved from https://icon.unicef.org/iconhome/Pages/FullStory.aspx?Title=LinkTitle&List=1699371f-2b32-4333-bdd7-6cc9397808b1&Fulltext=Full_x0020_Text_x0020_of_x0020_S&ItemID=1557.

[CR58] United Nations Children's Fund Nigeria & US Centers for Disease Control and Prevention (2015). Violence against children in Nigeria: findings from a national survey 2014.

[CR59] United Nations Children's Fund, US Centers for Disease Control and Prevention, & Kenya National Bureau of Statistics (2012). V*iolence against children in Kenya: Findings from a 2010 national survey*.

[CR60] United Nations Children's Fund, US Centers for Disease Control and Prevention, & Muhumbili University of Health and Allied Sciences. (2011). *Violence against children in Tanzania: findings from a national survey 2009.* Dar es Salaam: UNICEF.

[CR61] United Nations Economic and Social Council (2015). Country programme document: Indonesia.

[CR62] United Nations General Assembly (1989). Convention on the rights of the child.

[CR63] United Nations General Assembly (2015). Transforming our world: the 2030 agenda for sustainable development.

[CR64] Vagi K, Brookmeyer K, Gladden R, Chiang L, Brooks A, Nyunt M (2016). Sexual violence against female and male children in the United Republic of Tanzania. Violence Against Women.

[CR65] Veenema TG, Thornton CP, Corley A (2015). The public health crisis of child sexual abuse in low and middle income countries: an integrative review of the literature. International Journal of Nursing Studies.

[CR66] Watts C, Zimmerman C (2002). Violence against women: global scope and magnitude. The Lancet.

[CR67] World Health Organization (1999). Report of the consultation on child abuse prevention 29–31 march 1999.

[CR68] World Health Organization (2013). *Global and regional estimates of violence against women: prevalence and health effects of intimate partner violence and non-partner sexual violence*.

[CR69] World Health Organization (2014). Global status report on violence prevention 2014.

[CR70] World Health Organization (2014). Health for the world’s adolescents: a second chance in the second decade.

[CR71] Zimbabwe National Statistics Agency (ZIMSTAT), United Nations Children's Fund, & Collaborating Center for Operational Research (2013). National baseline survey on the life experiences of adolescents 2011.

[CR72] Zolotor A, Runyan D, Dunne M, Jain D, Peturs H, Ramirez C (2009). ISPCAN child abuse screening tool Children’s version (ICAST-C): instrument development and multi-national pilot testing. Child Abuse and Neglect.

